# Biventricular myocardial adaptation in patients with repaired tetralogy of Fallot: Mechanistic insights from magnetic resonance imaging tissue phase mapping

**DOI:** 10.1371/journal.pone.0237193

**Published:** 2020-08-11

**Authors:** Meng-Chu Chang, Ming-Ting Wu, Ken-Pen Weng, Kuang-Jen Chien, Chu-Chuan Lin, Mao-Yuan Su, Ko-Long Lin, Ming-Hua Chang, Hsu-Hsia Peng

**Affiliations:** 1 Department of Biomedical Engineering and Environmental Sciences, National Tsing Hua University, Hsinchu, Taiwan; 2 Department of Radiology, Kaohsiung Veterans General Hospital, Kaohsiung, Taiwan; 3 Faculty of Medicine, National Yang-Ming University, Taipei, Taiwan; 4 Department of Pediatrics, Kaohsiung Veterans General Hospital, Kaohsiung, Taiwan; 5 Department of Physical Therapy, Shu-Zen College of Medicine and Management, Kaohsiung, Taiwan; 6 Department of Medical Imaging, National Taiwan University Hospital, Taipei, Taiwan; 7 Department of Physical Medicine and Rehabilitation, Kaohsiung Veterans General Hospital, Kaohsiung, Taiwan; Faculty of Medical Science - State University of Campinas, BRAZIL

## Abstract

**Background:**

The myocardial adaptive mechanism in patients with repaired tetralogy of Fallot (rTOF) is less understood. We aimed to investigate biventricular myocardial adaptive remodeling in rTOF patients.

**Methods:**

We recruited 32 rTOF patients and 38 age- and sex-matched normal controls. The pulmonary stenosis of rTOF patients was measured using catheterized pressure gradient between right ventricle (RV) and pulmonary artery (PG_RVPA_). rTOF patients with PG_RVPA_ < 15 mmHg and ≥15 mmHg were classified as low pulmonary stenosis (rTOF_low_, n = 19) and high pulmonary stenosis (rTOF_high_, n = 13) subgroups, respectively. Magnetic resonance imaging tissue phase mapping was employed to evaluate the voxelwise biventricular myocardial motion in longitudinal (Vz), radial (Vr), and circumferential (Vφ) directions.

**Results:**

The rTOF_low_ subgroup presented higher pulmonary regurgitation fraction than rTOF_high_ subgroup (*p* < 0.001). Compared with the normal group, only rTOF_low_ subgroup presented a decreased RV ejection fraction (RVEF) (*p* < 0.05). The rTOF_low_ subgroup showed decreased systolic and diastolic Vz in RV and LV, whereas rTOF_high_ subgroup showed such change only in RV. In rTOF_low_ subgroup, RVEF significantly correlated with RV systolic Vr (r = 0.56, *p* < 0.05), whereas LVEF correlated with LV systolic Vz (r = 0.51, *p* = 0.02). Prolonged QRS correlated with RV systolic Vr (r = -0.58, *p* < 0.01) and LV diastolic Vr (r = 0.81, *p* < 0.001). No such correlations occurred in rTOF_high_ subgroup.

**Conclusions:**

The avoidance of unfavorable functional interaction in RV and LV in rTOF_high_ subgroup suggested that adequate pulmonary stenosis (PG_RVPA_ ≥ 15 mmHg in this sereis) has a protective effect against pulmonary regurgitation.

## Introduction

Pulmonary regurgitation (PR) is an important problematic sequel in patients after repair of tetralogy of Fallot (rTOF). Although PR can be tolerated for many years, chronic PR can lead to right ventricular (RV) volume overload, hypertrophy, fibrosis, myocardial injury, and heart failure overtime [1–4]. In addition, left ventricle (LV) remodeling is consequently affected [3,5–9]. Therefore, surgical strategy has shifted from complete relief of pulmonary stenosis (PS) toward restrictive enlargement of the pulmonary annulus to maintain a certain pressure gradient between the RV and pulmonary artery (PA) trunk (PG_RVPA_). Studies have observed that an adequate PG_RVPA_ can serve as a protective factor limiting the unfavorable effect of PR on RV function in rTOF patients [10–13].

Cardiac magnetic resonance imaging (MRI) is the standard method for comprehensive evaluation of rTOF patients [1–3,6,14]. MRI tissue phase mapping (TPM) has been approved for precise assessment of voxel-wise myocardial function for patients with a variety of heart diseases [15–19], and serves as a potential new diagnostic biomarker for rTOF patients [5]. As compared to speckle-tracking echocardiography and other MRI sequences for myocardial performance, such as myocardial tagging and feature tracking [20], TPM using a 2D acquisition has the advantages of less operator-dependence, high spatial resolution, and direct three-directional measurements of myocardial motion. Therefore, it simplifies the post-processing and enable LV as well as RV evaluation in rTOF.

In this case–control study, the catheterization-based PG_RVPA_ was measured to differentiate rTOF patients with low and high PS. We aimed to investigate the biventricular myocardial adaptive mechanisms on account of residual PS in rTOF patients using TPM from mechanistic insights.

## Methods

### Study cohort

This study protocol was approved by the ethics committee at Kaohsiung Veterans General Hospital, Kaohsiung (VGHKS14-CT1-16), Taiwan and all procedures were in accordance with the ethical standards of the institutional and research committees. All participants provided written informed consent prior to cardiac MRI examination. The study population consisted of 32 rTOF patients (age: 22.5 ± 3.8 years; male: 19) and 38 normal controls (age: 22.1 ± 1.8 years; male: 23) without known cardiovascular diseases. The rTOF patients underwent electrocardiography (ECG), treadmill, blood sampling for brain natriuretic peptide (BNP) measurement, and catheterization for assessment of hemodynamic status. The rTOF patients and normal controls were subjected to cardiac MRI examination. The datasets generated during and/or analyzed during this study are available from the corresponding author on reasonable request.

### Subgrouping of rTOF patients according to residual PS

Catheterization-based cutoff value of 15 mmHg based on PR of 40% in a receiver operating characteristic analysis was selected in this series. According to the cutoff value of PG_RVPA_, we defined rTOF patients with PG_RVPA_< 15 mmHg as the subgroup with low PS (rTOF_low_) and rTOF patients with PG_RVPA_ ≥ 15 mmHg as the subgroup with high PS (rTOF_high_). The surgical procedure of transannular patch was performed on 12 of 19 and 8 of 13 patients in rTOF_low_ and rTOF_high_ subgroups, respectively.

### Catheterization, cardiopulmonary exercise testing and laboratory testing

A 7 F Berman catheter was inserted into a femoral vein and a 5 F pig-tail catheter was inserted into the femoral artery when rTOF patients underwent catheterization. Both catheters were connected to pressure transducers. A Berman catheter was advanced into the right atrium, RV, and main PA to measure pressure. The PG_RVPA_ was also recorded. All pressures measurements were taken in the supine position and at end-expiration.The normal controls did not undergo catheterization.

The rTOF patients underwent an exercise stress test on a treadmill with the standard Bruce protocol [21]. An exercise testing equipment, which comprised a treadmill, a flow module, a gas analyzer, and an electrocardiographic monitor (Metamax 3B, Cortex Biophysik GmbH Co., Germany), was used to measure the exercise capacity. Peak oxygen consumption (VO_2_) was measured from the results of a graded treadmill exercise until exhaustion. Blood pressure (BP) and heart rate (HR) were also measured during resting and peak state. HR reserve (HRR) was defined as the HR change between 1 minute after test and peak state during test. The metabolic equivalent (MET) of peak VO_2_ was defined as the unit of resting oxygen uptake. The BNP level was measured using commercial ARCHITECT BNP Reagent Kits (Abbott Laboratories).

### Cardiac MRI acquisition

Images were acquired using a 3-T MR scanner (Skyra or Tim Trio, Siemens, Erlangen, Germany). A retrospective ECG-gating approach was used to acquire two-dimensional breath-hold steady-state free precession based cine images in short-axis view with 30 time frames per cardiac cycle. The protocol parameters were as follows: TR/TE = 3.1/1.6ms, pixel size = 1.17 × 1.17 mm^2^, slice thickness = 6 mm, interslice gap = 4mm, and flip angle = 50°. Consecutive 10–12 short-axis views covering the entire LV and RV enabled determination of the cardiac function, including the volumetric indices, mass, and ejection fraction (EF).

The two-dimensional phase-contrast MRI in this study was performed with retrospective ECG triggering and free breathing to calculate the PR fraction, which was defined as backward flow volume divided by forward flow volume. The scanning parameters were as follows: TR/TE = 9.9/2.7 ms, flip angle = 30°, matrix size = 192 × 174 (interpolated into 256 × 256), field of view = 24–32 cm, slice thickness = 6 mm, views per segment = 2, and average = 2. Forty phases per cardiac cycle were reconstructed. Velocity encoding was initially set at 150 cm/s and increased by 100 cm/s if an aliasing artifact was present. The main PA was targeted at its midpoint between the pulmonary valve and bifurcation. The imaging planes were prescribed as strictly perpendicular to the vessels by using the double-oblique technique.

A two-dimensional dark-blood fast low-angle shot sequence was performed to acquire TPM. Images were prescribed in consecutive three short-axis slices (base, mid, and apex). The basal slice was prescribed at 1cm beneath the mitral valve level at end-systole, followed consecutively by middle and apical slices. Prospective ECG-triggering was performed for synchronization with the cardiac motions. Navigator-echo monitoring was performed to trace the location of the right hemidiaphragm. Velocity encoding was set to 15 and 25 cm/s for in-plane and through-plane motions, respectively. The protocol parameters were as follows: TR/TE = 6.5/4.2 ms, pixel size = 1.17×1.17 mm^2^, slice thickness = 6 mm, flip angle = 7°, acceleration factor = 5 with the PEAK-GRAPPA accelerating technique [22], and temporal resolution = 26 ms. The total scanning time was approximately 6 minutes for the three slices.

### Cardiac MRI analysis

The myocardial motion was calculated using aninstitute-developed analysis tool written in MATLAB (Mathworks). After regions of interest had been determined manually on the magnitude images of each cardiac phase and each slice, the LV was divided into 16 segments according to American Heart Association recommendations [23], whereas the RV was divided into 10 segments for comprehension ofits regional motion [24]. We evaluated the peak myocardial motion in the systolic and diastolic phases in the longitudinal (Vz), radial (Vr), and circumferential (Vφ) directions as the quantitative indices of segmental and the global myocardial motion of the RV and LV. The diastolic Vφ referred to the second circumferential peak velocity during systolic period, as defined by Menza et al [24].

### Statistical analysis

The statistical significance of the difference between groups was assessed using ANOVA analysis or Fisher exact test when appropriate. The Pearson correlation coefficient was calculated for the relationship between any two interested parameters. Multiple comparisons using Bonferroni correction for the LV 16-segment and RV 10-segment model were performed to examine the significance of the altered myocardial motion. Receiver operating characteristic analysis was used to seek the cut-off value of the PG_RVPA_ for the classification of rTOF patients into rTOF_low_ and rTOF_high_ subgroups. Intra-observer and inter-observer variability was assessed in 10 rTOF patients and 10 normal controls. Inter-observer and intra-observer variability of TPM parameters was evaluated using the intraclass correlation coefficient (ICC). *P* < 0.05 was considered statistically significant.

## Results

### Demographic characteristics

Table 1 summarizes the demographic characteristics of the normal group, the rTOF group and the two rTOF subgroups. Both patient subgroups showed a lower peak VO_2_ than the regular standards reported by a previous study [8]. The other important data of rTOF patients during the exercise testing were as follows: resting systolic BP 124 ± 13 mmHg, resting diastolic BP 75 ± 7 mmHg, resting HR 81 ± 9 beats per minute, peak systolic BP 167 ± 20 mmHg, peak diastolic BP 82 ± 13 mmHg, peak HR 174 ± 14 beats per minute, and HRR 21 ± 7. All pressure indices of RV and PA in both rTOF_low_ and rTOF_high_ subgroups were substantially higher than the reported regular standards [25].

**Table 1:** Demographic characteristics and catheterization-based pressure measurements.

	Normal (n = 38)	rTOF (n = 32)	rTOF_low_ (n = 19)	rTOF_high_ (n = 13)	ANOVA *p* value	rTOF_low_ vs rTOF_high_ *p* value
Age (years)	22.1 ± 1.8	22.5 ± 3.8	22.4 ± 4.4	22.6 ± 2.9	0.77	0.88
Sex (male/female)	23/15	19/13	10/9	9/4	NA	NA
Height (cm)	169.1 ± 8.5	166.1 ± 9.0	165.3 ± 9.9	167.2 ± 7.8	0.30	0.57
Weight (kg)	61.7 ± 13.3	60.0 ± 12.5	58.4 ± 13.8	62.5 ± 10.5	0.60	0.37
BSA (m^2^)	1.7 ± 0.2	1.6 ± 0.2	1.6 ± 0.1	1.6 ± 0.1	0.32	0.37
BMI (kg/m^2^)	21.4 ± 3.7	21.7 ± 3.9	21.3 ± 4.6	22.2 ± 2.9	0.78	0.53
Systolic pressure (mmHg)	118.4 ± 11.4	118.8 ± 11.6	115.3 ± 9.7	124.7 ± 7.5	< 0.05	< 0.05
Diastolic pressure (mmHg)	72.4 ± 5.7	72.8 ± 6.2	71.2 ± 6.3	75.0 ± 5.6	0.20	0.23
HR (bpm)	74.2 ± 14.2	70.1 ± 7.9	66.4 ± 5.9	70.8 ± 11.1	0.43	0.27
QRS duration (ms)	82 ± 9^†^	142.8 ± 29.5	144.9 ± 33.3	139.6 ± 23.7	NA	0.62
Age at repair (years)	NA	3.0 ± 1.9	2.9 ± 2.2	2.4 ± 1.6	NA	NA
Transannular patch	0	20	12	8	NA	NA
NYHA I/II/III (a.u.)	38/0/0	14/18/0	8/11/0	6/7/0	NA	NA
Peak VO_2_ (MET)	9.9 ± 1.6^†^	8.0 ± 1.5	8.2 ± 1.4	7.7 ± 1.7	NA	0.24
BNP (pg/ml)	14.1 ± 12.4^†^	27.9 ± 17.5	30.7 ± 18.8	21.8 ± 13.3	NA	0.39
Pressure index (mmHg)						
RV sys. P	< 25‡	54.4 ± 20.7	46.7 ± 7.9	67.8 ± 28.9	0.01	0.01
RV dia. P	< 5‡	6.6 ± 4.2	6.8 ± 4.5	6.1 ± 3.8	0.72	0.71
RV mean P	5‡	15.1 ± 4.9	15.5 ± 5.4	14.3 ± 3.9	0.56	0.55
PA sys. P	< 25‡	39.0 ± 8.9	41.7 ± 8.8	33.2 ± 6.3	0.04	0.04
PA dia. P	< 5‡	13.7 ± 4.6	13.3 ± 5.3	14.5 ± 1.8	0.61	0.61
PA mean P	< 15‡	21.8 ± 5.2	22.6 ± 5.9	19.8 ± 2.7	0.25	0.25
PG_RVPA_	<5‡	17.5 ± 21.4	6.8 ± 5.3	33.1 ± 26.3	< 0.001	< 0.001

BSA: body surface area, BMI: body mass index, HR: heart rate, NYHA: New York Heart Association functional class, VO_2_: maximal oxygen consumption, BNP: brain natriuretic peptide. PG_RVPA_, pressure gradient between RV and pulmonary artery. rTOF_low_ and rTOF_high_ indicated rTOF subgroup with PG_RVPA_< 15 mmHg and ≥ 15 mmHg, respectively.

^†^Normal values from ^8^.

‡Normal standard from^25^. The *p* values in the far right column indicate the level of statistical significance between rTOF_low_ and rTOF_high_ subgroups.

### Global function of RV and LV

Table 2 outlines the cardiac magnetic resonance imaging measurements of the LV, RV, and PA. For the PA, both rTOF_low_ and rTOF_high_ subgroups presented significantly higher PR fractions than normal group (both *p* < 0.001). Moreover, PR fraction in rTOF_low_ subgroup was higher than that in rTOF_high_ subgroup (*p* < 0.001). Compared with normal controls, rTOF_low_ subgroup exhibited increases in most RV volumetric indices (all *p* < 0.001) and significant decreases in RVEF (*p* < 0.01). For the LV, the volumetric indices were similar in all groups, whereas the LV end systolic volume index decreased and LVEF increased only in rTOF_high_ subgroup (both *p* < 0.05).

**Table 2:** Cardiac magnetic resonance imaging measurements of both ventricles and the PA.

Parameter	Normal (n = 38)	rTOF (n = 32)	rTOF_low_ (n = 19)	rTOF_high_ (n = 13)	ANOVA *p* value	rTOF_low_ vs rTOF_high_ *p* value
LV						
LVESV (cm^3^)	30.1 ± 10.0	27.1 ± 10.8	29.1 ± 12.9	24.0 ± 5.4	0.21	0.55
LVEDV (cm^3^)	102.5 ± 22.1	96.3 ± 16.5	97.0 ± 18.6	95.2 ± 13.5	0.45	1.00
LVSV (cm^3^)	72.4 ± 14.1	70.6 ± 12.3	70.2 ± 14.4	71.1 ± 9.0	0.86	1.00
LVCI (L/min/m^2^)	2950.1 ± 545.1	3086.2 ± 835.4	3158.1 ± 932.1	2972.3 ± 677.2	0.57	1.00
LVM (g)	95.8 ± 23.9	89.0 ± 22.8	90.3 ± 23.7	86.8 ± 22.3	0.47	1.00
LVESVI (cm^3^/m^2^)	17.6 ± 5.5	16.3 ± 6.1	17.7 ± 7.1	14.1 ± 3.1*	0.17	0.29
LVEDVI (cm^3^/m^2^)	59.7 ± 10.7	58.4 ± 9.5	60.0 ± 11.1	56.1 ± 6.3	0.53	0.95
LVSVI (cm^3^/m^2^)	42.1 ± 5.9	42.8 ± 6.8	43.4 ± 8.3	41.9 ± 3.6	0.75	1.00
LVMI (g/m^2^)	55.6 ± 11.3	57.6 ± 14.8	54.8 ± 10.3	50.7 ± 9.6	0.41	0.93
LVEF (%)	70.9 ± 4.5	73.1 ± 5.4*	72.0 ± 6.3	74.9 ± 2.9*	0.06	0.32
LVPER (EDV/s)	-3.8 ± 0.9	-3.4 ± 0.5*	-3.3 ± 0.5	-3.6 ± 0.4	0.09	0.89
LVPFR (EDV/s)	5.3 ± 1.1	5.6 ± 1.3	5.6 ± 1.5	5.6 ± 1.0	0.53	1.00
RV						
RVESV (cm^3^)	57.9 ± 14.9	99.3 ± 57.0***	109.8 ± 65.3***	81.1 ± 34.2	< 0.001	0.16
RVEDV (cm^3^)	130.9 ± 28.7	195.4 ± 82.8***	210.4 ± 96.1***	169.6 ± 45.8	< 0.001	0.20
RVSV (cm^3^)	73.0 ± 16.6	96.1 ± 30.6***	100.5 ± 35.8***	88.5 ± 17.4	< 0.001	0.55
RVESVI (cm^3^/m^2^)	34.0 ± 7.5	60.2 ± 31.4***	67.2 ± 35.4***	48.2 ± 18.8*	< 0.001	0.06
RVEDVI (cm^3^/m^2^)	77.0 ± 13.5	119.0 ± 44.7***	129.1 ± 50.7***	101.6 ± 25.2	< 0.001	0.06
RVSVI (cm^3^/m^2^)	43.0 ± 8.1	58.7 ± 16.7***	61.9 ± 18.9***	53.5 ± 9.0*	< 0.001	0.21
RVEF (%)	55.9 ± 4.8	50.9 ± 9.5**	49.4 ± 9.7**	53.5 ± 9.0	< 0.01	0.41
PA						
PA max. area (mm^2^)	664.5 ± 92.6	981.2 ± 328.6***	1154.5 ± 309.4**	857.6 ± 335.7*	< 0.01	1.00
PA distensibility (a.u.)	0.5 ± 0.3	0.4 ± 0.2	0.4 ± 0.2	0.3 ± 0.1	0.16	0.85
PA net flow (L/cycle)	0.07 ± 0.01	0.06 ± 0.02	0.06 ± 0.02	0.06 ± 0.03	0.09	1.00
PR fraction (%)	1.7 ± 1.5	36.6 ± 17.1***	44.6 ± 11.9***	25.6 ± 17.5***	< 0.001	< 0.001

LVESVI/LVEDVI: left ventricular end-systole/end-diastolic volume index, LVSVI: LV stroke volume index, LVCI: LV cardiac index, LVMI: LV mass index, LVEF: LV ejection fraction, LVPER: LV peak ejection rate, LVPFR: LV peak filling rate, RVESV/RVEDV: right ventricular end-systole/end-diastolic volume, RVSVI: RV stroke volume index, RVEF: RV ejection fraction, PA: pulmonary artery, PR: pulmonary regurgitation.

*p < 0.05

**p < 0.01, and

***p < 0.001 indicate levels of statistical significance between the normal group and rTOF group/subgroups. The *p* values in the far right column indicate the level of statistical significance between rTOF_low_ and rTOF_high_ subgroups.

### Myocardial motion of RV and LV

**Fig 1:**
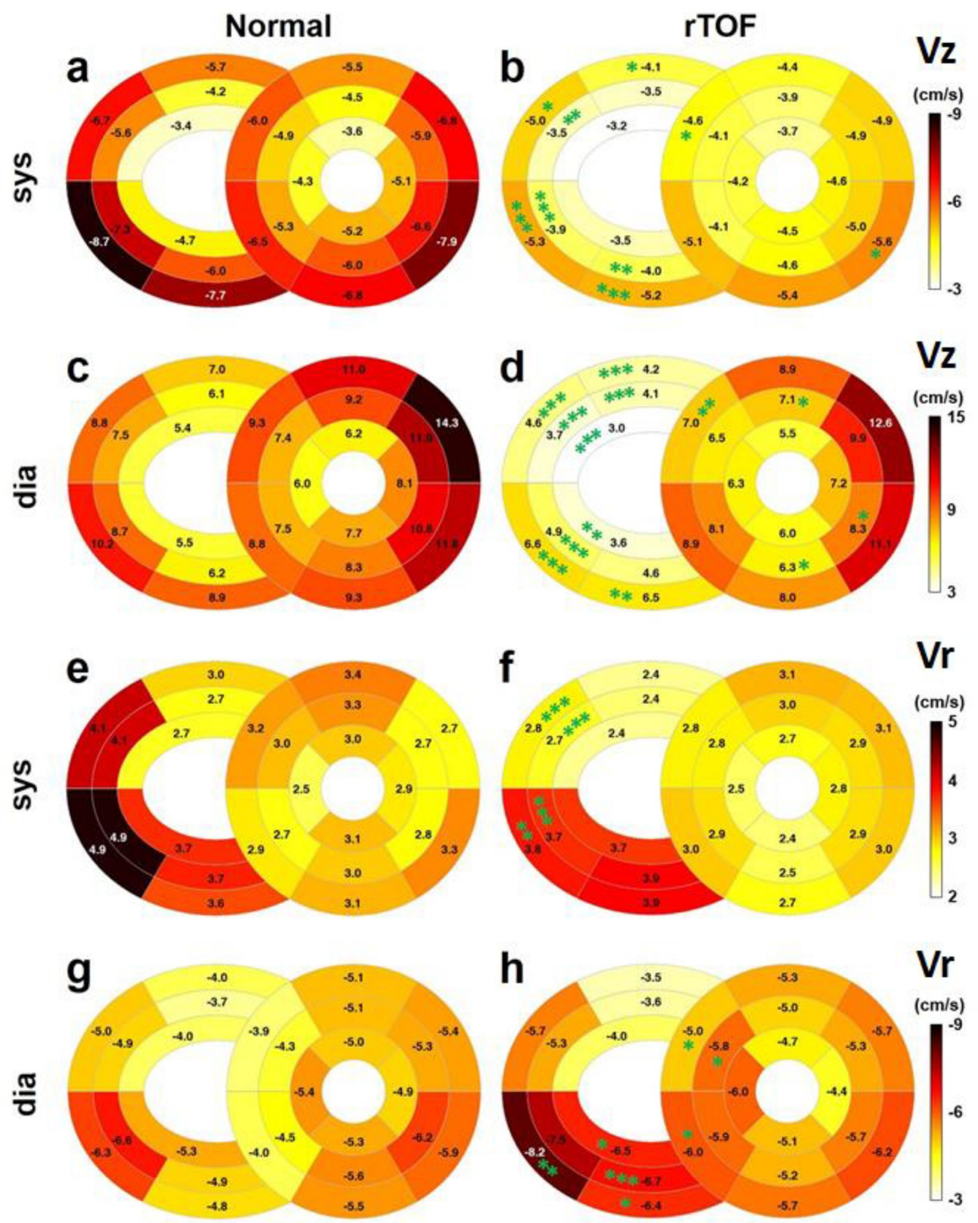
(a-d) The systolic and diastolic segmental myocardial motion in longitudinal (Vz) and (e–h) radial (Vr) directions in the normal controls (left panel) and patients with rTOF (right panel). *p < 0.05, **p < 0.01, and ***p < 0.001.

Fig 1 shows the segmental distribution of myocardial motion measured using TPM. Table 3 summarizes the global peak Vz, Vr, and Vφ of the 16 LV segments and 10 RV segments in each group. Compared with the normal group, the rTOF group showed significantly decreased RV systolic Vz (−4.0 ± 1.7 vs −6.0 ± 1.6 cm/s, *p* < 0.001), particularly in the basal and middle slices (Fig 1). The rTOF group also showed lower RV diastolic Vz in almost all RV segments (4.5 ± 1.4 vs 7.4 ± 1.5 cm/s, *p* < 0.001) and lower LV diastolic Vz (7.9 ± 1.8 vs 9.2 ± 2.0 cm/s, *p* < 0.05). Regarding the radial myocardial motion, only the RV diastolic Vr in the rTOF group was higher than that in the normal group (−5.7 ± 1.0 vs −4.9 ± 0.8 cm/s, *p* < 0.01). Decreased systolic and diastolic Vφ (i.e. the second peak during systole) were noted only in the LV of rTOF patients (*p* < 0.001). All the aforementioned indices did not differ significantly between rTOF_low_ and rTOF_high_ subgroups.

**Table 3:** Mean TPM derived measurements of global intramural motion in both ventricles.

	Normal (n = 38)	rTOF (n = 32)	rTOF_low_ (n = 19)	rTOF_high_ (n = 13)	rTOF_low_ vs rTOF_high_ *p* value
LV					
sys. Vz (cm/s)	-5.6 ± 1.7	-4.6 ± 1.5**	-4.2 ± 1.6**	-5.0 ± 1.2	0.16
dia. Vz (cm/s)	9.2 ± 2.0	7.9 ± 1.8*	7.6 ± 1.7**	8.5 ± 2.0	0.24
sys. Vr (cm/s)	2.9 ± 0.4	2.8 ± 0.4	2.7 ± 0.4	2.9 ± 0.2	0.37
dia. Vr (cm/s)	-5.1 ± 0.6	-5.4 ± 0.6	-5.4 ± 0.7	-5.4 ± 0.6	0.77
sys. Vφ (cm/s)	-3.4 ± 1.3	-2.0 ± 1.1***	-1.7 ± 1.1***	-2.0 ± 1.1***	0.13
dia. Vφ (cm/s)	1.8 ± 0.8	0.9 ± 0.5***	0.8 ± 0.5***	0.9 ± 0.5***	0.11
RV					
sys. Vz (cm/s)	-6.0 ± 1.6	-4.0 ± 1.7***	-3.6 ± 1.7***	-4.8 ± 1.7*	0.08
dia. Vz (cm/s)	7.4 ± 1.5	4.5 ± 1.4***	4.2 ± 1.4***	5.0 ± 1.3***	0.12
sys. Vr (cm/s)	3.1 ± 0.8	3.0 ± 0.6	2.9 ± 0.5	3.1 ± 0.8	0.85
dia. Vr (cm/s)	-4.9 ± 0.8	-5.7 ± 1.0**	-5.6 ± 0.7**	-5.7 ± 1.4*	0.80
sys. Vφ (cm/s)	-3.3 ± 1.3	-3.0 ± 1.3	-3.1 ± 1.5	-2.9 ± 1.1	0.45
dia. Vφ (cm/s)	2.7 ± 1.3	1.9 ± 0.8	2.0 ± 0.9	1.8 ± 0.8	0.53

Dia.: diastolic; LV: left ventricle; RV: right ventricle; sys.: systolic; TPM: tissue phase mapping. The value of these indices were averaged from 16 segments in the LV or 10 segments in the RV. The diastolic Vφ was the second circumferential velocity peak during systolic period, as defined in reference [25]. rTOF_low_ and rTOF_high_ indicated rTOF subgroup with PG_RVPA_ < 15 mmHg and ≥ 15 mmHg, respectively.

*p < 0.05

**p < 0.01, and

***p < 0.001 indicate levels of statistical significance between the normal group and rTOF group/subgroups. The *p* values in the far right column indicate the level of statistical significance between the two rTOF subgroups.

### Effect of PR on RV pressure and biventricular function in rTOF subgroups

**Fig 2:**
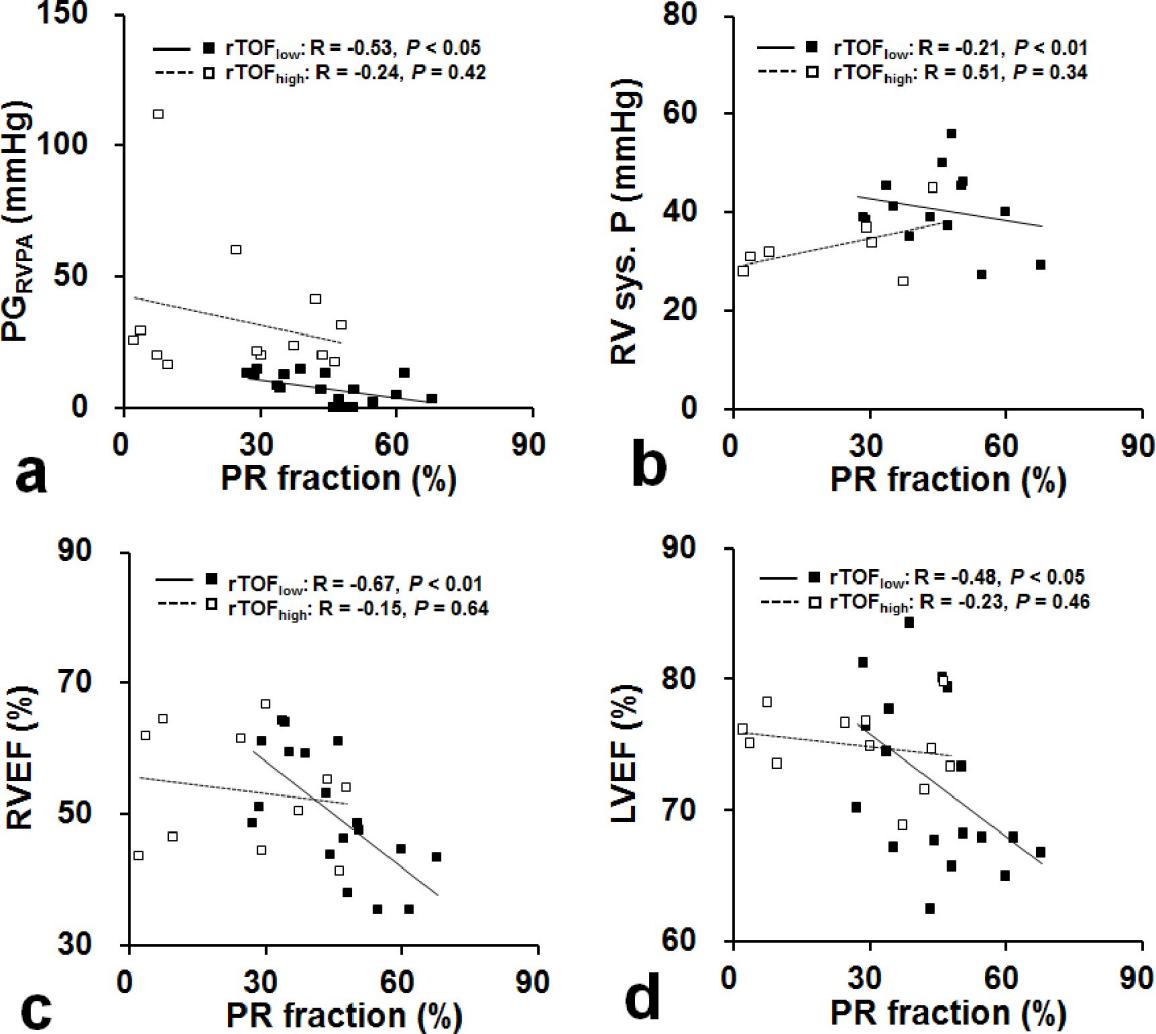
Scatterplots of PR fraction vs. PG_RVPA_ (a), RV systolic pressure (b), RVEF (c), and LVEF (d). Significant correlations existed only in rTOF with low pulmonary stenosis.

In rTOF_low_ subgroup, the PR fraction showed significantly negative correlations with PG_RVPA_ (r = −0.53, *p* < 0.05; Fig 2A) and RV systolic pressure (r = −0.21, *p* < 0.01; Fig 2B). Moreover, the PR fraction negatively correlated with the RVEF (r = −0.67, *p* < 0.01; Fig 2C) and LVEF (r = −0.48, *p* < 0.05; Fig 2D) only in rTOF_low_ subgroup.

### Differential relationship of myocardial motion and electromechanical adaption in RV and LV

**Fig 3:**
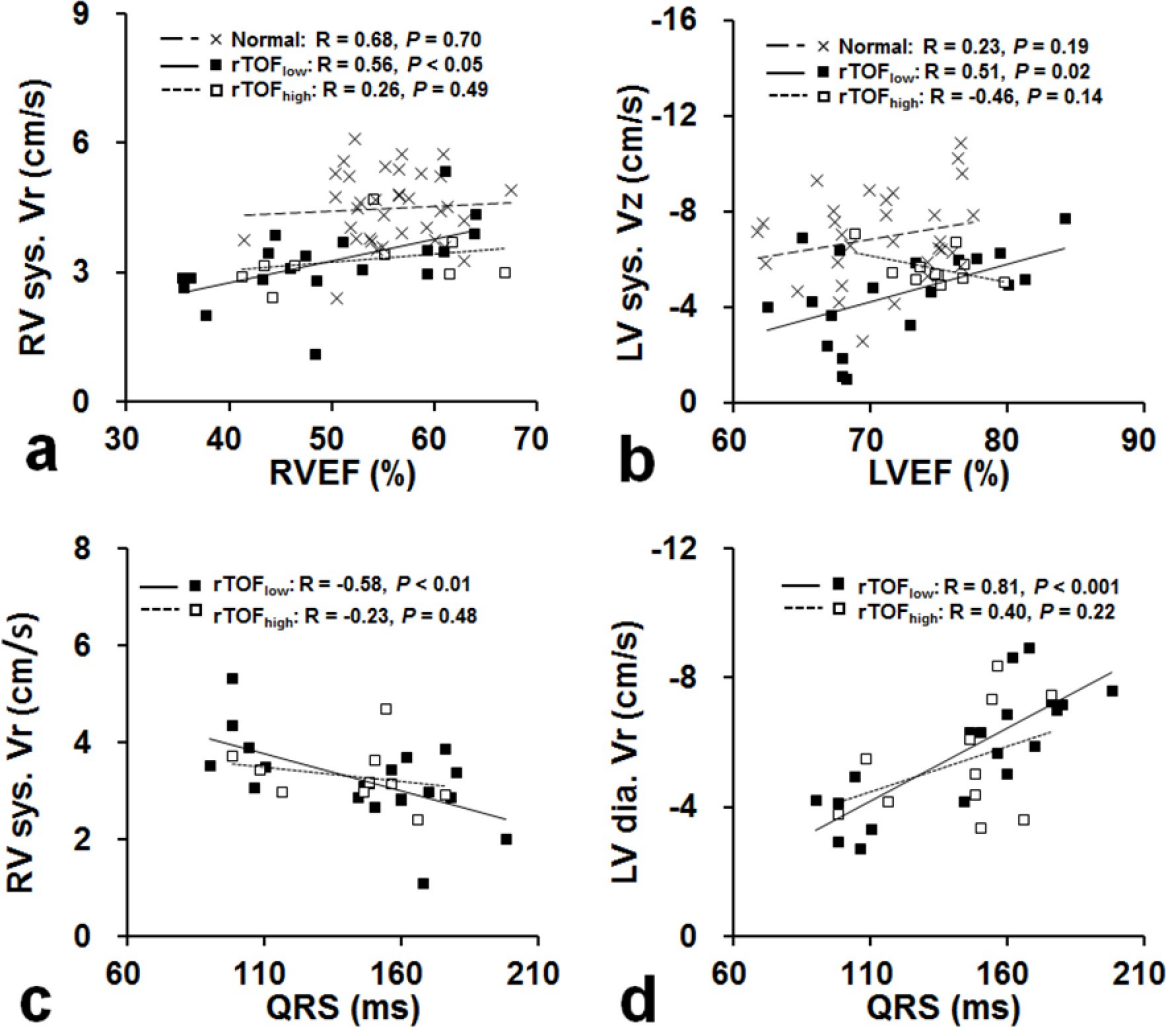
Scatterplots of RVEF vs. RV systolic Vr (a), LVEF vs. LV systolic Vz (b), QRS vs. RV systolic Vr (c) and QRS vs. LV diastolic Vr (d). Significant correlations existed only in rTOF with low pulmonary stenosis.

In rTOF_low_ subgroup, RVEF had a significant positive correlation with RV systolic Vr (r = 0.56, *p* < 0.05; Fig 3A), whereas LVEF had a significant positive correlation with LV systolic Vz (r = 0.51, *p* = 0.02; Fig 3B).

Compared with reference values in Table 1, QRS durations in both rTOF_low_ and rTOF_high_ subgroups were substantially prolonged. There was no significant difference between rTOF_low_ and rTOF_high_ subgroups in terms of QRS duration (144.9 ± 33.3 vs 139.6 ± 23.7 ms, *p* = 0.62). However, only QRS duration in rTOF_low_ subgroup was significantly negatively correlated with RV systolic Vr (r = −0.58, *p* < 0.01; Fig 3C) and positively correlated with LV diastolic Vr (r = 0.81, *p* < 0.001; Fig 3D). No other significant findings or correlations were observed in rTOF_high_ subgroup or normal group.

### Interventricular correlation between RV and LV functions

**Fig 4:**
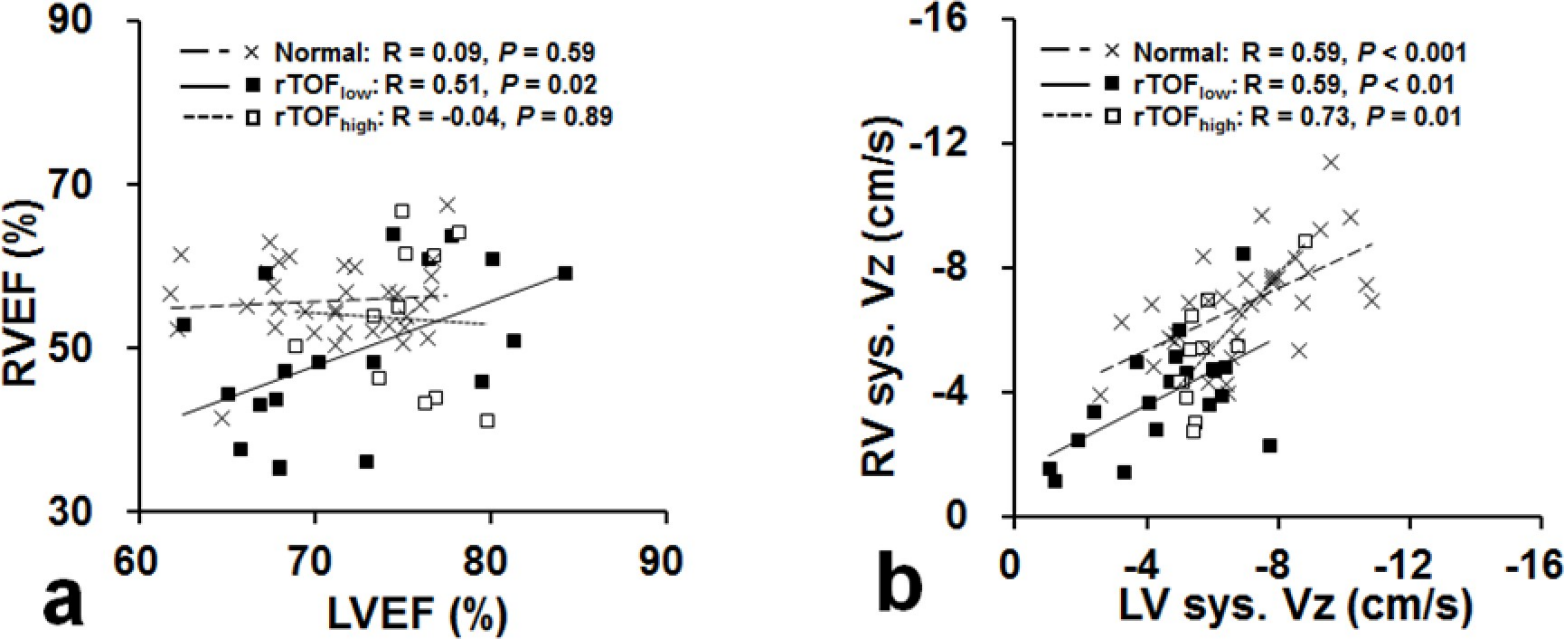
Correlation between RV and LV in (a) EF and (b) systolic longitudinal motion (sys. Vz).

The RVEF and LVEF were significantly correlated only in the rTOF_low_ subgroup (Fig 4A). However, myocardial motion of systolic Vz between RV and LV was significantly correlated in rTOF_low_ subgroup, rTOF_high_ subgroup, and normal group (all *p* ≤ 0.01; Fig 4B).

### Inter-observer and intra-observer variability of TPM parameters

The inter-observer ICC for TPM parameters was 96%, while the intra-observer ICC for TPM parameters was 95%.

## Discussion

In this study, we employed TPM and catheterization-based PG_RVPA_ to elucidate the biventricular adaptive mechanism in rTOF patients. Our study demonstrated that PR fraction plays a key role in the adaptive remodeling of RV and LV in rTOF patients. Our application of TPM also revealed that the adaption of differential myocardial motions correlated with the electromechanical remodeling of both RV and LV. Notably, these findings were almost observed solely in the rTOF_low_ subgroup. Avoidance of interconnected relationships among PR and biventricular myocardial function was found in rTOF_high_ subgroup, and this suggests that adequate residual PS may have a protective effect against PR related biventricular dysfunction in rTOF patients.

Significant negative correlations between PR fraction and RVEF (or LVEF) were observed solely in rTOF_low_ subgroup. Such adverse effects of PR on RVEF and LVEF were not seen in rTOF_high_ subgroup, suggesting that an adequate PG_RVPA_ may be beneficial for attenuating the vicious cycle triggered by PR in rTOF. Our findings are in line with the concept that adequate residual PS might be a suitable surgical strategy for treating TOF [10–13] and resulted in better biventricular remodeling.

Valente et al previously reported that PR and RV volume were not related to early death or ventricular tachycardia in rTOF patients [26]. However, their study group [26] included 36% patients with pulmonary valve replacement and 14% with RV-PA conduit whose results should not be generalized to rTOF patients with native PR in the current study. Compared to the possible pressure overestimation of previous echocardiography-derived cutoff values varying from 20 to 30 mmHg [10–13], catheterization-based cutoff value of 15 mmHg based on PR of 40% in a receiver operating characteristic analysis was objectively selected in this series. On the other hand, although our results demonstrated the potential association between PS and myocardial adaptation in young adult rTOF patients in a relatively early stage without showing clinical adverse outcomes, the potential harm of PS on ventricular function can not be neglected according to Valente et al’s study [26]. The appropriate PS in rTOF patients with various clinical conditions requires further investigations in a large cohort.

TPM has the advantages of high spatial resolution and direct three-directional measurements of myocardial motion. This technique has been used to demonstrate that rTOF patients may exhibit RV myocardial motion abnormality owing to excess stress in response to volume overload [1,6,14]. We found that most myocardial motion in rTOF_low_ subgroup decreased in both RV and LV, whereas myocardial motion in rTOF_high_ subgroup decreased in RV and was normal in LV. This may suggest that LV adaption was secondary to RV changes with the progression of RV dysfunction.

We found that differential myocardial motion adaption underlined RV and LV remodeling in rTOF_low_ subgroup. RVEF closely correlated with RV systolic Vr, whereas LVEF closely correlated with LV systolic Vz. The discrepancy between RV and LV adaption may be partially explained by the different inherent but integrative myofiber architectures in the two ventricles and also by a substantial adaptive increased thickness of the circumferential layer found histopathologically in the RV of rTOF patients [27,28]. In addition, RV diastolic Vr paradoxically increased in rTOF group, which may reflect a compensatory radial motion accelerated predominantly in the diastolic phase of overloaded RV [7].

We found prolonged QRS durations in both rTOF subgroups. Electromechanical interaction with prolonged QRS duration has been described as a predictor of ventricular arrhythmias in rTOF patients [4,29,30]. However, QRS prolongation was positively correlated with LV diastolic Vr and inversely with RV systolic Vr only in the rTOF_low_ subgroup. These findings suggested that altered myocardial radial motion, either in RV or LV, may underscore the adverse effect of QRS prolongation in rTOF patients without protection from adequate residual PS [4,9,31].

In a scintigraphy study of 152 patients, Movahed et al demonstrated that there was a strong significant correlation between LVEF and RVEF in patients with decreased EF, but no correlation was found in patients with normal EF [32]. This phenomenon was replicated in our results. In normal subjects and rTOF_high_ patients, RVEF and LVEF are both preload- and afterload-dependent, and thus are not tied physiologically. The presence of biventricular EF correlation in rTOF_low_ subgroup may imply an unfavorable effect of RV on LV, and thus may also reflect the deteriorated cardiac function in rTOF patients without adequate PS. The myocardial longitudinal motion of RV and LV was disclosed to be significantly correlated in all subjects, independent of the rTOF status. The role of myocardial longitudinal motion correlation between RV and LV requires future investigation.

Although we showed the decreased LV Vφ during systole in rTOF patients compared with that of normal controls, it should be noted that the assessment of circumferential myocardial motion was more difficult to identify compared with longitudinal and radial motion [5,24]. Further, because of the complicated and low diastolic myocardial motion velocity in rTOF patients, a simple measurement of Vφ might not be able to illustrate the difference between the groups. A previous study calculated the twist function by systolic peak-to-peak Vφ and reported the difference of LV circumferential motion between normal and rTOF groups [5]. However, the twist function of RV is more complicated and required designation of a novel and accurate approach. We were unable to report RV twist function because of the limited scale in this study.

In conclusion, this study explored the adaptation of biventricular function and the differential myocardial motion components in rTOF patients in response to residual PS. From a mechanistic insight of myocardial motion adaptation, avoidance of unfavorable functional interaction in RV and LV in rTOF_high_ subgroup suggested that adequate PS (PG_RVPA_ ≥ 15 mmHg in this series) has a protective effect against PR in the ventricular remodeling of rTOF. Our findings shed light on the mechanical and electrical adaptation in both RV and LV of rTOF patients and potentially provide helpful information for surgical strategy in treating patients with TOF. A larger and longer cohort study is required to investigate the prognostic value of our findings in evaluation of rTOF patients.

### Limitations

This study had some limitations. First, the thin wall of RV may have rendered the reliability of RV delineation in the TPM images. However, we acquired TPM images with a pixel size of 1.17 × 1.17 mm^2^, which was consistent with the spatial resolution of a highly ranked previous study on TPM of RV [33]. Second, RV twist function was not calculated and we may have oversimplified the complex biventricular adaptive mechanism in rTOF patients. Third, this cross-sectional study involved a relatively low sample size, a short postoperative period, and a lack of events such as pulmonary valve replacement and pacemaker implantation. Fourth, there is no T1 mapping data to evaluate cardiac fibrosis. A larger cohort and longer follow-up may further elucidate and validate the role of PR and PS on account of myocardial motion adaption in the prognosis of rTOF.

## Supporting information

S1 File (DOCX)
