# Supplementary material for: Biventricular myocardial adaptation in patients with repaired tetralogy of Fallot: Mechanistic insights from magnetic resonance imaging tissue phase mapping
Source: PLoS One. 2020 Aug 11;15(8):e0237193. doi: 10.1371/journal.pone.0237193 (PMC7418997; doi:10.1371/journal.pone.0237193)
Supplement: S1 File — (DOCX) [file pone.0237193.s001.docx]

1. Clarify the peak VO2 value for controls: Peak VO_2_ in normal controls is 9.9 ± 1.6 Met. (p29 last line in Table 1)
2. Other important measures of the CEPT: The other important data of rTOF patients during the exercise testing were as follows: resting systolic BP 124 ± 13 mmHg, resting diastolic BP 75 ± 7 mmHg, resting HR 81 ± 9 beats per minute, peak systolic BP 167 ± 20 mmHg, peak diastolic BP 82 ± 13 mmHg, peak HR 174 ± 14 beats per minute, and HRR 21 ± 7. (p7 last line to p8 line 6)
3. Table 3 is included in the revised manuscript. (p34-35)

**Table 3.** Mean TPM derived measurements of global intramural motion in both ventricles.

|  | **Normal**  **(n=38)** | | **rTOF**  **(n=32)** | **rTOF_low_**  **(n=19)** | | **rTOF_high_**  **(n=13)** | | **rTOF_low_ vs rTOF_high_**  ***p* value** |
| --- | --- | --- | --- | --- | --- | --- | --- | --- |
| LV |  |  | |  |  | |  | |
| sys. Vz (cm/s) | -5.6 ± 1.7 | | -4.6 ± 1.5** | -4.2 ± 1.6** | | -5.0 ± 1.2 | | 0.16 |
| dia. Vz (cm/s) | 9.2 ± 2.0 | | 7.9 ± 1.8* | 7.6 ± 1.7** | | 8.5 ± 2.0 | | 0.24 |
| sys. Vr (cm/s) | 2.9 ± 0.4 | | 2.8 ± 0.4 | 2.7 ± 0.4 | | 2.9 ± 0.2 | | 0.37 |
| dia. Vr (cm/s) | -5.1 ± 0.6 | | -5.4 ± 0.6 | -5.4 ± 0.7 | | -5.4 ± 0.6 | | 0.77 |
| sys. Vφ (cm/s) | -3.4 ± 1.3 | | -2.0 ± 1.1*** | -1.7 ± 1.1*** | | -2.0 ± 1.1*** | | 0.13 |
| dia. Vφ (cm/s) | 1.8 ± 0.8 | | 0.9 ± 0.5*** | 0.8 ± 0.5*** | | 0.9 ± 0.5*** | | 0.11 |
| RV |  | |  |  | |  | |  |
| sys. Vz (cm/s) | -6.0 ± 1.6 | | -4.0 ± 1.7*** | -3.6 ± 1.7*** | | -4.8 ± 1.7* | | 0.08 |
| dia. Vz (cm/s) | 7.4 ± 1.5 | | 4.5 ± 1.4*** | 4.2 ± 1.4*** | | 5.0 ± 1.3*** | | 0.12 |
| sys. Vr (cm/s) | 3.1 ± 0.8 | | 3.0 ± 0.6 | 2.9 ± 0.5 | | 3.1 ± 0.8 | | 0.85 |
| dia. Vr (cm/s) | -4.9 ± 0.8 | | -5.7 ± 1.0** | -5.6 ± 0.7** | | -5.7 ± 1.4* | | 0.80 |
| sys. Vφ (cm/s) | -3.3 ± 1.3 | | -3.0 ± 1.3 | -3.1 ± 1.5 | | -2.9 ± 1.1 | | 0.45 |
| dia. Vφ (cm/s) | 2.7 ± 1.3 | | 1.9 ± 0.8 | 2.0 ± 0.9 | | 1.8 ± 0.8 | | 0.53 |

Dia.: diastolic; LV: left ventricle; RV: right ventricle; sys.: systolic; TPM: tissue phase mapping. The value of these indices were averaged from 16 segments in the LV or 10 segments in the RV. The diastolic Vφ was the second circumferential velocity peak during systolic period, as defined in reference [25]. rTOF_low_ and rTOF_high_ indicated rTOF subgroup with PG_RVPA_ < 15 mmHg and ≥ 15 mmHg, respectively. **p* < 0.05, ***p* < 0.01, and ****p* < 0.001 indicate levels of statistical significance between the normal group and rTOF group/subgroups. The *p* values in the far right column indicate the level of statistical significance between the two rTOF subgroups.

1. Additional data of echocardiographic PG between RV and PA and correlation data with PG_RVPA_ from catheterization: In our rTOF patients, PG_RVPA_ (echocardiography) was 21.5 ± 16.6 mmHg. PG_RVPA_ (catheterization) was 17.5 ± 21.4 mmHg. There was a high correlation between PG_RVPA_ (echocardiography) and PG_RVPA_ (catheterization) (r = 0.925, p < 0.001). Pressure overestination of echocardiography derived value by 8 mmHg was noted.
